# Robotic-Assisted Bronchoscopy for Peripheral Pulmonary Lesions: Current Evidence, Clinical Applications, and Future Directions

**DOI:** 10.3390/diagnostics16193151

**Published:** 2026-09-28

**Authors:** Venkatkiran Kanchustambham

**Affiliations:** 1 Interventional Pulmonology and Cystic Fibrosis Program, Sanford Health, Fargo, ND 58102, USA; vkanchus@icloud.com; 2 Department of Internal Medicine, University of North Dakota School of Medicine and Health Sciences, Grand Forks, ND 58203, USA

**Keywords:** robotic-assisted bronchoscopy, peripheral pulmonary lesions, lung nodule, Ion endoluminal system, monarch platform, galaxy system, cone-beam CT, diagnostic yield, cryobiopsy, CT-to-body divergence, navigational bronchoscopy, lung cancer screening, transbronchial ablation, molecular profiling, interventional pulmonology

## Abstract

The evaluation of peripheral pulmonary lesions (PPLs) is a persistent clinical challenge, made more pressing by the expansion of low-dose CT lung cancer screening. Conventional and earlier navigational bronchoscopy, including electromagnetic navigation, historically achieved diagnostic yields of approximately 60–70%, with CT-to-body divergence contributing to nondiagnostic sampling. This narrative review, prepared using the Scale for the Assessment of Narrative Review Articles (SANRA), summarizes robotic-assisted bronchoscopy (RAB) platform technology, procedural optimization, diagnostic performance, safety, molecular tissue adequacy, and emerging applications. A structured search was updated through September 2026; this is not a systematic review. Using the strict definition recommended by the American Thoracic Society/American College of Chest Physicians (ATS/ACCP), the most comprehensive meta-analysis reported a pooled RAB diagnostic yield of 69.6%, while the prospective multicenter TARGET study reported an adjudicated strict yield of 61.6%. Higher pooled estimates of approximately 84–87% generally use intermediate definitions that the ATS/ACCP statement recommends against as primary diagnostic-yield measures. RAB pneumothorax rates are approximately 2% in pooled analyses, compared with approximately 20–25% for CT-guided transthoracic needle biopsy. In the randomized RELIANT trial, RAB was non-inferior, but not superior, to electromagnetic navigation bronchoscopy; in VERITAS, navigational bronchoscopy as a category was non-inferior to transthoracic needle biopsy for 12-month diagnostic accuracy, with fewer pneumothoraces. RAB is a useful option for selected PPLs, but strict-definition performance is more modest than frequently quoted intermediate yields. Outcomes depend on lesion characteristics, sampling strategy, operator experience, center volume, and imaging infrastructure.

## 1. Introduction

Robotic-assisted bronchoscopy (RAB) has emerged as an important advancement in the diagnostic evaluation of peripheral pulmonary lesions (PPLs), addressing fundamental limitations of conventional bronchoscopy and earlier navigational platforms. The timely and accurate tissue diagnosis of PPLs is central to lung cancer management, particularly following the 2021 US Preventive Services Task Force expansion of low-dose CT screening eligibility, which lowered the age threshold to 50 years and the qualifying smoking history to 20 pack-years, substantially increasing the estimated eligible screening population [1].

Historically, the diagnostic evaluation of peripheral lung nodules relied on two primary modalities. CT-guided transthoracic needle biopsy (TTNB) achieves diagnostic yields of 80–90% for accessible peripheral lesions but carries pneumothorax rates of 20–25% and chest-tube requirements of 7–8% [2]. Conventional flexible bronchoscopy offers a favorable safety profile but has limited peripheral yield; pooled and meta-analytic data show substantially reduced yields for small (<2 cm) peripheral lesions [3,4], and the AQuIRE registry reported a yield of 63.7% for nonguided bronchoscopy [5]. Electromagnetic navigation bronchoscopy (ENB) improved peripheral navigational access, yet data from the prospective NAVIGATE study demonstrated a diagnostic yield of only 67.8% at 24 months [6]. This ceiling has been attributed primarily to CT-to-body divergence (CTBD), the systematic displacement of lesion position between the static preprocedural CT scan and the dynamic intraoperative pulmonary environment [7].

Although PPLs are the principal focus of this review, small or anatomically difficult central and mid-zone lesions may also be challenging and are not categorically excluded; lesion size and accessibility, rather than location alone, determine procedural difficulty.

RAB integrates robotic catheter control with advanced navigation technologies—shape-sensing fiber-optic localization or electromagnetic guidance—and compatibility with intraprocedural imaging adjuncts, principally cone-beam computed tomography (CBCT), to improve lesion localization and procedural stability. Early prospective studies and meta-analyses suggest improved diagnostic performance compared with conventional bronchoscopic techniques [8,9,10,11,12,13] and a favorable safety profile compared with transthoracic approaches, particularly in patients at elevated pneumothorax risk. However, outcomes vary meaningfully with lesion characteristics, operator experience, institutional volume, and availability of adjunctive imaging. Rigorous interpretation of the evidence therefore requires careful attention to diagnostic-yield definitions, study design, and procedural heterogeneity [8,9,10].

This review provides an evidence-based synthesis of RAB encompassing platform technology, preprocedural planning, diagnostic performance, adjunctive imaging strategies, biopsy optimization, safety, comparative effectiveness, advanced applications, training, cost-effectiveness, and future directions. The literature search strategy, inclusion criteria, and synthesis approach are described in Section 2. As lung cancer screening expands, optimizing minimally invasive diagnostic strategies for peripheral lesions remains a priority.

## 2. Methods: Literature Search Strategy and Study Selection

This narrative review was prepared using the Scale for the Assessment of Narrative Review Articles (SANRA; checklist in Supplementary Table S1), which supports transparent reporting and critical appraisal of narrative reviews but does not replace systematic-review methods or formal risk-of-bias assessment. No protocol was registered because this is a narrative rather than a systematic review. The search strategy, inclusion criteria, and synthesis approach were specified before literature retrieval, and the search was updated to include relevant publications available through September 2026.

Because this narrative review does not apply formal evidence grading, the patient-selection and ventilatory-strategy tables in Section 5 deliberately avoid recommendation grading. Instead, they separate the clinical scenario or procedural strategy from the certainty of the supporting evidence, described qualitatively as moderate, low, or very low according to study design and directness. Most supporting evidence is observational or indirect, and entries resting on expert consensus or mechanistic reasoning are identified as such. These certainty descriptors reflect confidence in the underlying evidence only and do not constitute graded recommendation.

### 2.1. Search Strategy

A structured literature search was conducted across four electronic databases—PubMed/MEDLINE, Embase, Web of Science, and the Cochrane Library—for articles published from January 2018 (the year the first RAB platform received FDA clearance) through September 2026. Controlled-vocabulary terms (MeSH/Emtree) and free-text keywords were combined using Boolean operators (AND/OR). Primary terms included “robotic bronchoscopy,” “robotic-assisted bronchoscopy,” “Ion endoluminal system,” “Monarch platform,” “Galaxy system,” “navigational bronchoscopy,” and “shape-sensing bronchoscopy.” These were combined with secondary terms: “peripheral pulmonary lesion,” “pulmonary nodule,” “diagnostic yield,” “cone-beam CT,” “CBCT,” “CT-to-body divergence,” “cryobiopsy,” “transbronchial cryobiopsy,” “next-generation sequencing,” “molecular profiling,” “lung cancer screening,” “transbronchial ablation,” “learning curve,” and “cost-effectiveness.” A supplementary search of ClinicalTrials.gov and the WHO International Clinical Trials Registry Platform (ICTRP) identified ongoing and recently completed trials. Conference abstracts from the American Thoracic Society (ATS; 2022–2025), CHEST (2022–2025), the European Respiratory Society (ERS; 2022–2025), and the American Association for Bronchology and Interventional Pulmonology (AABIP; 2022–2025) were manually screened to capture relevant data available only in abstract form, including the ERS 2025 randomized controlled trial by Steinack and colleagues and the October 2025 FDA clearance of the Ion artificial intelligence navigational system.

### 2.2. Inclusion and Exclusion Criteria

Studies were included if they evaluated robotic-assisted bronchoscopy platforms (Ion, Monarch, or Galaxy) in human subjects and reported diagnostic yield, safety outcomes, molecular tissue adequacy, or clinical effectiveness endpoints. Conference abstracts presented at major society meetings were included when no full-text publication was available and the data materially contributed to the evidence synthesis; such abstracts are identified as non-peer-reviewed where cited. Historical context regarding the evolution of navigational bronchoscopy (ENB, radial endobronchial ultrasound [R-EBUS], and conventional bronchoscopy) was drawn from landmark studies, meta-analyses, and registry datasets without restriction to the 2018–2026 window. Preclinical animal studies were excluded from the evidence synthesis but are cited where they provide the only available safety data for an emerging therapeutic application, such as transbronchial ablation. Cadaveric proof-of-concept studies were retained only when clearly identified and when they materially informed platform feasibility or comparative technical performance. Other exclusions were (1) case reports with fewer than five subjects, unless the reported application was novel with no other available human data, and (2) editorials or letters without original data.

### 2.3. Study Selection and Data Synthesis

Literature screening was performed by the author in two stages: title and abstract screening followed by full-text review of potentially eligible articles. A narrative synthesis was used rather than formal meta-analytic pooling; quantitative estimates from published meta-analyses are cited with attention to definition, design, and heterogeneity. Evidence included meta-analyses, prospective multicenter studies, randomized trials, registries, retrospective cohorts, conference abstracts, guidelines, consensus statements, and expert reviews. Because screening and study selection were conducted by a single reviewer without duplicate adjudication or formal risk-of-bias assessment, selection and confirmation bias remain possible. SANRA was used to improve reporting quality, not to imply systematic-review-level bias control.

## 3. Historical Context: The Evolution of Peripheral Bronchoscopy

Throughout this review, “conventional (nonguided) flexible bronchoscopy” refers to flexible bronchoscopy performed with standard-diameter scopes under fluoroscopic guidance only, without electromagnetic or shape-sensing navigation. This is explicitly distinct from thin and ultrathin bronchoscopy and from radial-EBUS–assisted sampling, each of which is treated as a separate modality where cited. Unless otherwise specified, all subsequent references to “conventional bronchoscopy” carry this definition.

Early bronchoscopy, pioneered by Gustav Killian in 1897 for foreign body retrieval, used rigid instruments that provided central airway access only (Figure 1). The development of flexible fiberoptic bronchoscopy by Shigeto Ikeda in 1966 extended reach into segmental and subsegmental airways, enabling bronchial washings, brushings, and transbronchial forceps biopsies. However, diagnostic yield for subcentimeter or peripheral lesions remained severely limited by the inability to navigate beyond the bronchoscopist’s direct field of view.

Fluoroscopy-guided transbronchial biopsy was the first attempt to extend diagnostic bronchoscopy to radiographically visible but endoscopically inaccessible lesions. Systematic reviews demonstrated reasonable sensitivity (~88%) for central lesions but only ~34% yield for peripheral lesions under 2 cm [19]. R-EBUS, first described in 1992, added real-time confirmation of probe position within or adjacent to peripheral lesions, modestly improving the pooled yield to approximately 71% [4].

Electromagnetic navigation bronchoscopy (ENB), commercialized by the SuperDimension platform in 2004, was the watershed technology bringing navigation-assisted peripheral bronchoscopy into mainstream practice. ENB uses an electromagnetic field generator to track a sensor probe within a registered CT-derived airway map. Real-world performance was constrained by CTBD: Pritchett and colleagues characterized this displacement as typically 5–15 mm, sufficient to cause the navigation system to confirm position within the lesion on its virtual map while the biopsy tool is displaced from the actual lesion in real space [7]. The NAVIGATE trial, enrolling 1215 patients across 37 centers, reported a 24-month diagnostic yield of only 67.8% [6].

Ultrathin bronchoscopes (outer diameter 2.8 mm) improved access to more distal airways but did not address CTBD. The field recognized that a step-change was needed—one providing structural stability, precise distal articulation, real-time lesion localization beyond navigation mapping alone, and reliable tissue acquisition. This recognition set the stage for the introduction of robotic platforms in the late 2010s.

## 4. Robotic-Assisted Bronchoscopy Platforms: Technology and Engineering

Three robotic-assisted bronchoscopy systems are currently FDA-cleared and in clinical use (Table 1). While all three share the overarching goal of improving navigational precision and catheter stability for PPL biopsy, they differ substantially in technical architecture, navigation methodology, scope design, and imaging integration. Recent reviews summarize these platform-level distinctions and evolving clinical applications [20].

### 4.1. Ion™ Endoluminal System (Intuitive Surgical)

The Ion endoluminal system, FDA-cleared in February 2019, is widely deployed in clinical practice [14]. Ion’s most distinctive feature is its proprietary shape-sensing technology: a fiber-optic network with Bragg gratings embedded within the catheter wall that measures the catheter’s three-dimensional shape and precise position hundreds of times per second, enabling real-time location awareness entirely independent of electromagnetic fields. This eliminates interference from metallic equipment and obviates the need for external electromagnetic field generators.

The Ion catheter has a 3.5 mm outer diameter, enabling navigation to distal airways, commonly around the 6th–7th generation, though reachable depth varies with airway caliber and patient anatomy and more distal access is achievable. The fully articulating distal tip provides 180-degree deflection in any plane. All three platforms are additionally compatible with R-EBUS, fluoroscopy, CBCT or digital tomosynthesis, and the 1.1 mm cryoprobe, and the Ion catheter provides notable positional stability during sampling. Pre-procedural planning uses PlanPoint software, which automatically segments the target nodule from CT imaging and generates navigation pathways with vascular proxies defining anatomic danger zones. In October 2025, Intuitive received FDA clearance for AI integration across Ion’s complete navigational workflow—the first AI-embedded bronchoscopy navigation system—a development expected to further reduce learning curve variability [18].

### 4.2. Monarch™ Platform (Johnson & Johnson/Auris Health)

The Monarch Platform, FDA-cleared in March 2018, was the first RAB system to achieve commercial availability [15]. Monarch combines electromagnetic navigation with optical and robotic-kinematic position data and uses a distinctive telescoping dual-scope design: an outer guiding sheath (6.0 mm outer diameter) and an inner steerable bronchoscope (4.2 mm outer diameter) with a built-in camera, allowing continuous white-light visualization of the peripheral airway during navigation and biopsy. The BENEFIT feasibility study (*n* = 54) reported R-EBUS-confirmed lesion localization in 96.2% of cases [15]. The TARGET trial was a prospective, multicenter, single-arm study of 679 patients (median lesion size 1.85 cm) that reported an adjudicated strict diagnostic yield of 61.6% using ATS/ACCP criteria, a sensitivity for malignancy of 78.8%, and an adverse-event rate of 3.8% [21].

### 4.3. Galaxy System™ (Noah Medical)

The Galaxy System, FDA-cleared in March 2023, is the newest entrant and incorporates several distinctive innovations [16]. Its most notable feature is integrated Tool-in-Lesion Tomosynthesis (TiLT+), a proprietary digital tomosynthesis system that provides intraoperative three-dimensional imaging for real-time confirmation of biopsy-tool position within the target lesion, addressing CTBD without requiring a separate CBCT unit. Galaxy uses electromagnetic guidance to reach within approximately 2 cm of the lesion and then activates TiLT for final localization with an augmented fluoroscopy overlay. The Galaxy bronchoscope is single-use and disposable, eliminating reprocessing logistics; comparative per-case cost data remain limited. The first-in-human FRONTIER study (19 nodules in 18 patients; mean lesion size 2.0 cm) reported 100% TiLT-confirmed tool-in-lesion localization, with diagnostic yields of 89.5% (strict) and 94.7% (intermediate) [16]. A multicenter trial (NCT06056128) is ongoing.

## 5. Preprocedural Planning, Patient Selection, and Procedural Setup

### 5.1. CT Imaging Requirements

Optimal RAB outcomes require high-quality thin-slice (≤1 mm collimation) chest CT in breath-hold. Thicker reconstructions significantly impair automated airway segmentation accuracy. The CT is imported into platform-specific planning software (PlanPoint for Ion; Monarch Planning Software; Galaxy Planning Software), which segments the bronchial tree from the trachea through sub-subsegmental airways. Key planning parameters include: depth of airway pathway (generations to target), bronchus sign presence, lesion-to-pleura distance, vascular proximity, and anticipated need for CBCT adjunct. The bronchus sign—defined as a bronchus leading directly into or through the lesion on CT—remains an important predictor of diagnostic success, though its impact is attenuated by CBCT-guided tool-in-lesion confirmation [8,9].

### 5.2. Patient Selection

Patient selection for RAB should be individualized based on lesion characteristics, patient risk profile, availability of institutional expertise, and whether the clinical question requires tissue for diagnosis, molecular profiling, or both. Table 2 summarizes evidence-based indications, preferred scenarios, and contraindications in full detail. The following provides a structured clinical framework.

#### 5.2.1. Primary Indications

RAB may be considered for (1) PPLs, most commonly in the outer two-thirds of the lung though not categorically limited to this region, where conventional flexible bronchoscopy has limited diagnostic yield (14% for peripheral lesions <2 cm; 31% for inner two-thirds lesions [22]); (2) intermediate- to high-probability pulmonary nodules, defined by Lung-RADS category 4A/4B [26] or by an intermediate-to-high malignancy probability on the Mayo model (5–65%), that require tissue diagnosis per American College of Chest Physicians (ACCP) and National Comprehensive Cancer Network (NCCN) guidelines [24,25]; (3) suspected or confirmed non-small cell lung cancer (NSCLC) requiring comprehensive molecular profiling, including a next-generation sequencing (NGS) panel, programmed death-ligand 1 (PD-L1) immunohistochemistry (IHC), ALK/ROS1 fluorescence in situ hybridization (FISH), and tumor mutational burden (TMB), particularly when liquid biopsy is insufficient or preserved tissue architecture is advantageous for IHC interpretation [27,28]; and (4) peripheral lesions with concurrent fluorodeoxyglucose (FDG)-avid mediastinal or hilar lymphadenopathy, where combined peripheral biopsy and endobronchial ultrasound-guided transbronchial needle aspiration (EBUS-TBNA) staging in a single procedure is clinically appropriate per ACCP/NCCN guidelines [24,25], including selected station 5/6 approaches reported with robotic bronchoscopy [29,30,31].

#### 5.2.2. Potential Advantages over CT-Guided Transthoracic Needle Biopsy

RAB may be favored over CT-TTNB in selected patients when the transthoracic approach carries elevated risk or is technically limited: (1) emphysema or bullous lung disease, in which CT-TTNB pneumothorax risk is 20–25% overall and substantially higher when emphysematous parenchyma overlies the biopsy path [2], whereas RAB avoids pleural traversal and has a pooled pneumothorax rate of approximately 2.0% [8,10]; (2) a single functional lung or severely impaired contralateral pulmonary reserve, in which a pneumothorax could have major clinical consequences; (3) thrombocytopenia (platelet count <50,000/µL), coagulopathy (international normalized ratio [INR] >1.5), or therapeutic anticoagulation that cannot be safely interrupted, in which TTNB hemorrhage risk is elevated [2]; (4) deep-seated lesions requiring long parenchymal traversal (>3 cm), vascular proximity, or prior ipsilateral pleurodesis limiting lung mobility [2]; and (5) bilateral synchronous nodules requiring same-session evaluation, for which bilateral percutaneous sampling is generally avoided because of bilateral pneumothorax risk [32,33].

#### 5.2.3. Lung Cancer Screening Population

Low-dose CT (LDCT)-detected nodules classified as Lung-RADS 4A or 4B [26] represent a growing indication for RAB. The 2021 US Preventive Services Task Force (USPSTF) expansion of screening eligibility to age 50 years and 20 pack-years substantially increased the eligible population [1], generating a large volume of screen-detected nodules that require risk stratification and, in many cases, tissue evaluation. Pooled RAB diagnostic yield is approximately 78% for lesions <2 cm, the size range of many LDCT-detected nodules, although this estimate uses non-strict definitions [9]. The favorable safety profile of RAB may be particularly relevant in this population of current and former smokers, in whom emphysema is common.

#### 5.2.4. Relative and Absolute Contraindications

Relative contraindications include very small lesions (<6–8 mm), which are lower-yield but not automatically excluded; CBCT-assisted series have reported yields of approximately 66.6% for lesions ≤10 mm, and the decision should depend on whether a biopsy result would change management [36,37,38]. Pure ground-glass opacities may have lower cellular yield, and Fleischner Society or Lung-RADS [26] surveillance recommendations should guide whether tissue sampling is indicated. Inability to tolerate general anesthesia is another relative contraindication, although moderate sedation may be considered for selected cases with favorable anatomy at experienced centers. Absolute contraindications include active bronchospasm or status asthmaticus and hemodynamic instability or acute respiratory failure requiring escalating support. Uncorrectable severe coagulopathy contraindicates cryobiopsy specifically; standard fine-needle aspiration (FNA) or forceps biopsy may remain feasible after appropriate correction.

### 5.3. Anesthetic and Ventilatory Optimization

RAB is generally performed under general anesthesia with endotracheal intubation and neuromuscular blockade at high-volume centers, distinguishing it from conventional bronchoscopy under moderate sedation. General anesthesia provides airway control and limits patient movement during navigation, allows controlled ventilation and PEEP to reduce intraoperative atelectasis, and accommodates the 45–90 min duration of complex cases [41]. The I-LOCATE trial established that significant atelectasis develops as early as 30 min post-intubation, a critical time benchmark for procedural planning [42]. Comprehensive ventilatory strategies to optimize RAB outcomes are detailed in Table 3.

## 6. Diagnostic Yield: Evidence Base and Key Determinants

Diagnostic yield is the primary outcome metric for PPL bronchoscopy, but comparisons require attention to the definition used. The evidence below therefore focuses on RAB meta-analyses, prospective trials, adjunctive imaging, and sampling technique; the historical ~70% ceiling and the CTBD mechanism are summarized in Section 3.

### 6.1. Meta-Analytic Evidence for RAB

The most defensible headline estimates are those using the strict definition recommended by the American Thoracic Society (ATS)/ACCP (Figure 2). Li and colleagues [10] reported a pooled strict RAB diagnostic yield of 69.6% (95% CI 61.8–76.8%) across 27 cohort studies, with pneumothorax in 2.0% and chest-tube placement in 0.5%. TARGET, the largest prospective multicenter single-platform RAB study, reported an adjudicated strict yield of 61.6% [21]. Higher estimates should be interpreted according to their definitions: Ali and colleagues [8] reported an intermediate yield of 84.3% (95% CI 81.1–87.2%), and Li and colleagues reported 86.6% using an intermediate definition [10]. The ATS/ACCP statement recommends against intermediate and liberal definitions because they incorporate subsequent clinical information or follow-up into the index-test result [45]. Zhang and colleagues [9] reported a pooled yield of 80.4%, with higher yields in studies using cryobiopsy, but heterogeneity in study design and yield definitions limits cross-study comparison. A 2026 clinical review in the *New England Journal of Medicine* similarly noted that TARGET’s approximately 62% strict yield was not clearly better than that of other guided bronchoscopy techniques and characterized the evidence for real-time localization adjuncts as mixed [46]. Key prospective studies, randomized trials, and meta-analyses are summarized in Table 4, and additional prospective shape-sensing RAB series are summarized in Table 5.

### 6.2. Pivotal Prospective Trials

Among platform-specific prospective data, TARGET [21] (described in Section 4.2) reported an adjudicated strict diagnostic yield of 61.6% using ATS/ACCP criteria in 679 patients. The single-center RELIANT cluster-randomized non-inferiority trial [49] compared RAB directly with digital-tomosynthesis-corrected ENB in 411 patients; diagnostic yield, defined as the proportion of cases yielding lesional tissue, was 77.8% with RAB and 75.5% with ENB, meeting the prespecified non-inferiority criterion (*p* = 0.007) without demonstrating superiority. VERITAS [17], a multicenter randomized non-inferiority trial of navigational bronchoscopy broadly rather than RAB alone, enrolled 234 patients with nodules 10–30 mm and reported 12-month diagnostic accuracy of 79.0% with navigational bronchoscopy versus 73.6% with CT-guided TTNB. Pneumothorax occurred in 3.3% versus 28.3%, and chest-tube placement or hospital admission in 0.8% versus 11.5%, respectively. These trials support bronchoscopic-first strategies in appropriately selected patients but do not establish superiority of RAB over ENB or CT-guided biopsy.

### 6.3. Diagnostic Yield Definitions: ATS/ACCP 2024 Consensus

Diagnostic yield is the proportion of bronchoscopic procedures that produce a specific diagnosis from the index procedure under a prespecified definition. Diagnostic accuracy additionally requires verification against a reference standard over follow-up, as in VERITAS. The 2024 ATS/ACCP Research Statement [45] recommends a strict diagnostic-yield definition based on a specific malignant or benign diagnosis established by the bronchoscopic sample and recommends against so-called intermediate or liberal definitions, which can incorporate follow-up or management decisions into the index-test result. Accordingly, this review treats strict yield as the primary benchmark and presents intermediate values only as secondary historical estimates. TARGET’s adjudicated strict yield was 61.6% [21].

### 6.4. Key Predictors of Diagnostic Success

Predictors of higher RAB diagnostic yield identified across multiple studies and meta-analyses include lesion size greater than 20 mm, presence of a CT bronchus sign, a concentric R-EBUS view confirming tool position within the lesion, CBCT-confirmed tool-in-lesion, and center or operator experience [8,9,10,59]. Predictors of lower yield include pure ground-glass or subsolid density, absence of a bronchus sign, upper-lobe location, lesion depth beyond the sixth airway generation, and lesion size below 10 mm [8,9,10]. CBCT-assisted Ion studies have reported yields of approximately 66.6% for lesions ≤10 mm [36,37,38].

## 7. Adjunctive Imaging: Overcoming CT-to-Body Divergence

CT-to-body divergence (CTBD) represents a fundamental limitation of navigation-based bronchoscopy, reflecting the spatial discrepancy between pre-procedural CT imaging—acquired in a breath-holding, awake, supine patient—and intraoperative lung anatomy under general anesthesia with controlled mechanical ventilation. Factors contributing to CTBD include atelectasis onset (significant atelectasis develops within approximately 30 min of intubation per the I-LOCATE trial [42]), gravitational redistribution of lung tissue, altered lung volumes under positive pressure ventilation, and diaphragmatic displacement. Resulting lesion displacement is typically 5–15 mm in magnitude per Pritchett and colleagues [7], sufficient to cause systematic biopsy of peritumoral or atelectatic tissue while the actual lesion remains unsampled, even when the navigation system confirms apparent tool-in-lesion on the virtual map. CTBD is the primary explanatory variable for the persistent gap between navigational success rates (often >95%) and actual diagnostic tissue acquisition in ENB and early RAB series.

### 7.1. Cone-Beam CT: Fixed and Mobile Systems

CBCT provides intraoperative three-dimensional volumetric imaging that can show biopsy-tool position relative to the lesion independently of the preprocedural CT-derived navigation map (Figure 3). Both fixed and mobile systems, including the Cios Spin (Siemens Healthineers, Erlangen, Germany), have been evaluated. In the prospective Bashour study [36] (*n* = 67; lesions 9–30 mm), tool-in-lesion confirmation increased from 34.3% before mobile CBCT to 98.6% after imaging and repositioning (absolute improvement 64.3 percentage points; *p* < 0.0001). This finding demonstrates improved localization but should not automatically be interpreted as an equivalent increase in diagnostic yield. Evidence for a yield benefit is mixed and may depend on platform, workflow, operator experience, and case selection. In a 2026 retrospective Monarch study, strict diagnostic yield was 70.9% without mobile CBCT and 71.7% with mobile CBCT (*p* = 0.976), while radiation dose increased from 7.4 to 285.9 mGy [50]. CBCT availability is also limited by capital cost, workflow complexity, radiation exposure, and infrastructure requirements.

### 7.2. Randomized Evidence for RAB + CBCT

A randomized comparison of RAB + CBCT with ultrathin bronchoscopy under two-dimensional fluoroscopy was presented at the 2025 ERS Congress by Steinack and colleagues [40]. As of the September 2026 search update, these data remained available only as a non-peer-reviewed conference abstract. The abstract reported a diagnostic yield of 84.6% with RAB + CBCT versus 23.1% with the comparator (78 patients; 127 PPLs; median lesion size 11 mm; absolute difference 61.5 percentage points; 95% CI 44.1–78.9; *p* < 0.001). Overall, 85% of lesions lacked a CT bronchus sign and 27.6% were pure ground-glass opacities. Because the report is preliminary, single-center, and not peer-reviewed, it should not be weighted equivalently to published multicenter trials or used as a primary headline estimate.

### 7.3. Radial EBUS, Digital Tomosynthesis, and Augmented Fluoroscopy

Radial EBUS (R-EBUS) remains the most widely available intraoperative lesion-localization adjunct, providing real-time 360-degree cross-sectional ultrasound confirmation that the probe is within or adjacent to the target lesion. A concentric R-EBUS view is consistently associated with higher diagnostic yield across studies and meta-analyses [4]. Digital tomosynthesis (TiLT+ technology, Galaxy System; Illumisite, Medtronic, Minneapolis, MN, USA), offers an alternative approach to CTBD correction using standard C-arm fluoroscopic data, without the infrastructure requirements of CBCT [16]. Augmented fluoroscopy overlays the lesion’s intraoperative position onto real-time fluoroscopy and may permit tool repositioning. Multimodal localization is associated with high yields in experienced-center series, but the incremental diagnostic benefit of CBCT is not uniform across platforms or centers [36,37,38,50].

## 8. Biopsy Technique Optimization and Molecular Adequacy

### 8.1. Rationale for Cryobiopsy

Conventional biopsy tools passed through the 2.0 mm working channel of the Ion catheter, such as 21/22-gauge FNA needles and standard forceps, have inherent limitations (Table 6). FNA yields small, often fragmented cytological specimens that lack architectural detail, and forceps biopsies are susceptible to crush artifact and may inadequately sample eccentric lesions that lie adjacent to, rather than around, the airway. These tool limitations may contribute to nondiagnostic sampling. Cryobiopsy exploits the Joule–Thomson effect: rapid expansion of compressed gas at the probe tip cools it to approximately −79 °C with CO_2_ (or lower with N_2_O), freezing adjacent tissue within seconds. Unlike forceps, the 1.1 mm cryoprobe acquires tissue circumferentially, which is potentially advantageous for eccentric lesions that abut but do not surround the airway [27,60].

### 8.2. Clinical Evidence

Oberg and colleagues [27] retrospectively analyzed 112 patients with 120 PPLs sampled during Ion RAB with sequential FNA, forceps, and 1.1 mm cryoprobe biopsy. Overall yield was 90%, 18% of diagnoses were made exclusively by cryobiopsy, and all cryobiopsy samples submitted for molecular testing were adequate. A 2024 retrospective cohort comparing cryobiopsy with FNA also reported favorable cryobiopsy performance in small nodules [62]. Randomized evidence has since strengthened the broader bronchoscopic cryobiopsy literature. FROSTBITE-2 [63], a multicenter trial that randomized 500 patients with lung nodules or masses, lung transplants, or diffuse parenchymal lung disease, reported an overall diagnostic yield of 88.6% with the 1.1 mm cryoprobe versus 78.8% with forceps, and 83.2% versus 70.1% in the nodule-or-mass subgroup. FROSTBITE-2 did not identify a safety disadvantage with the cryoprobe: pneumothorax requiring chest-tube placement occurred in four patients in the forceps arm and none in the cryoprobe arm, and neither arm had grade 3–4 bleeding. In the PPL-specific Cryo-RCT [60], cryobiopsy improved histology-based yield overall (83% vs. 75%) and for adjacent or eccentric R-EBUS views (77% vs. 65%), but moderate bleeding (38% vs. 19%) and pneumothorax (2% vs. <1%) were more frequent. Safety findings therefore differ between the two trials. Neither trial was restricted to robotic bronchoscopy, and neither evaluated a complete RAB + CBCT strategy.

### 8.3. Molecular Adequacy for Next-Generation Sequencing

Contemporary management of lung adenocarcinoma requires not only a tissue diagnosis but also comprehensive molecular profiling, including broad-panel NGS, PD-L1 IHC, and companion diagnostic assays. Inadequate tissue quantity or quality may prevent patients from receiving effective targeted therapies. The American Association for Bronchology and Interventional Pulmonology–International Association for the Study of Lung Cancer (AABIP-IASLC) clinical practice guideline [64] concluded that guided bronchoscopy achieves tissue adequacy for comprehensive biomarker testing comparable to that of CT-guided percutaneous biopsy (80–100% vs. 72.3–96%). Whether adding CBCT and cryobiopsy to RAB further improves adequacy has not yet been established.

In a Mayo Clinic study, Yu Lee-Mateus and colleagues [61] found that 72% of 78 RAB samples met preanalytic criteria for molecular or PD-L1 testing; NGS adequacy was 48.6% for smears, 61.4% for touch preparations, and 29.2% for biopsies. In a Memorial Sloan Kettering series, Connolly and colleagues [28] reported that 84% of 128 RAB samples were adequate for molecular testing, with success rates of 96% (25/26) for hybrid-capture NGS, 94% (49/52) for polymerase chain reaction (PCR)-based testing, and 91% for PD-L1 IHC. Many experienced centers therefore use multimodal sampling, combining FNA, forceps biopsy, and cryobiopsy, with rapid on-site evaluation (ROSE), to maximize diagnostic yield and molecular adequacy, although no combination has been shown to be superior in a randomized trial.

## 9. Safety Profile and Complication Management

RAB’s safety profile is an important clinical advantage relative to CT-guided TTNB (Table 7) [8,10,21]. Across meta-analyses, pooled pneumothorax rates are approximately 2.0–2.3%, with chest-tube placement in 0.5–1.2%, compared with approximately 20–25% pneumothorax and 7–8% chest-tube placement in broader TTNB datasets [2]. In VERITAS, the randomized TTNB pneumothorax rate was higher at 28.3%, with chest-tube placement or admission in 11.5%, compared with 3.3% and 0.8%, respectively, after navigational bronchoscopy [17]. The overall pooled RAB complication rate in the 2025 meta-analysis was 3.0% [10], and TARGET reported a 3.8% adverse-event rate with no procedure-related deaths [21]. Cryobiopsy can increase tissue yield but may carry a bleeding and pneumothorax trade-off, as observed in Cryo-RCT [60].

Pneumothorax risk is increased by emphysematous lung parenchyma, upper lobe location, pleural proximity, multiple biopsy passes, and periprocedural movement. In patients with severe emphysema—precisely those at highest TTNB risk—RAB provides a compelling alternative enabling tissue acquisition with acceptable complication probability. The emerging literature suggests CT-guided TTNB may carry a risk of pleural track tumor seeding in confirmed malignancies—a theoretical risk not associated with airway-based bronchoscopic approaches. Rare complications have also been reported and warrant awareness, including pneumatocele formation following RAB [65], pneumomediastinum after transbronchial cryobiopsy [66], and systemic or cerebral arterial air embolism complicating robotic-guided lung biopsy [67]; although individually uncommon and described in case reports, these underscore the importance of meticulous technique and vigilance for air embolism under positive-pressure ventilation.

Radiation exposure from CBCT depends on the equipment, number of spins, acquisition protocol, and procedural workflow. Dose-optimization protocols should be used, and spins should be limited to those needed for localization or repositioning. Radiation should not be characterized as uniformly modest: in a 2026 Monarch comparison, median radiation dose was 285.9 mGy with mobile CBCT versus 7.4 mGy without it, approximately a 40-fold difference, without a significant diagnostic-yield improvement [50].

## 10. Comparative Effectiveness: RAB Versus Competing Modalities

### 10.1. RAB vs. CT-Guided Transthoracic Needle Biopsy

CT-guided TTNB achieves high diagnostic performance for accessible peripheral lesions but carries substantially greater pneumothorax risk. In VERITAS, a randomized non-inferiority trial [17], navigational bronchoscopy achieved 12-month diagnostic accuracy of 79.0% versus 73.6% with TTNB (absolute difference 5.4 percentage points; 95% CI −6.5 to 17.2; *p* = 0.003 for non-inferiority). Pneumothorax occurred in 3.3% versus 28.3%, and chest-tube placement or hospital admission occurred in 0.8% versus 11.5%. VERITAS evaluated the broader category of navigational bronchoscopy rather than RAB specifically and was conducted at expert centers, limiting direct extrapolation to every RAB program. A propensity score-matched study by Yu Lee-Mateus and colleagues [68] found that patients evaluated with RAB were more likely to receive an early-stage lung cancer diagnosis than those evaluated with TTNB (OR 3.02; 95% CI 1.83–5.04; *p* < 0.001), although this observational association should not be interpreted as proof that RAB caused stage migration. For subsolid nodules, Fernandez-Bussy and colleagues [69] reported comparable diagnostic yields with fewer complications in the RAB group.

### 10.2. RAB vs. Electromagnetic Navigation Bronchoscopy

In RELIANT [49], RAB was non-inferior but not superior to digital-tomosynthesis-corrected ENB (diagnostic yield, defined as lesional tissue obtained, 77.8% vs. 75.5%; Section 6.2). Subgroup findings, including those for smaller lesions and lesions without a CT bronchus sign, should be considered exploratory and should not be used to infer a causal advantage from robotic maneuverability. The imaging context also matters: the ENB comparator incorporated digital tomosynthesis correction, whereas CBCT images in the RAB arm were not transmitted to the navigation platform. A propensity score-matched analysis by McNierney and colleagues [70] found comparable yields between Monarch and Ion, suggesting that adjunctive imaging, sampling strategy, case selection, and operator experience may be at least as important as platform choice.

### 10.3. Clinical Integration and Modality Selection

Selection of the appropriate diagnostic modality for peripheral pulmonary lesions should be individualized based on lesion characteristics, patient risk profile, and institutional resources and expertise. CT-guided TTNB remains an effective and appropriate option for pleural-based or easily accessible peripheral lesions, particularly in settings where advanced bronchoscopic technologies are not available or where patient anatomy renders bronchoscopic access impractical. Continued imaging surveillance rather than biopsy is appropriate when a histologic result would not change management (e.g., very small or pure ground-glass lesions per Fleischner/Lung-RADS), and surgical or single-anesthetic diagnostic-resection pathways may be preferable for high-probability early-stage lesions in operable candidates.

Bronchoscopic approaches, including RAB, may be favored when (1) concurrent mediastinal staging is indicated, enabling diagnosis and staging in a single procedure per ACCP/NCCN guidelines [24,25]; (2) CT-guided biopsy carries elevated pneumothorax risk because of overlying emphysema, bullous disease, or deep parenchymal traversal [2]; (3) bilateral synchronous nodules require same-session evaluation [32,33]; (4) coagulopathy contraindicates the transthoracic approach; or (5) combined diagnosis and resection in a single anesthetic event is being considered [51].

In practice, RAB may be combined with radial EBUS, CBCT where available, and complementary biopsy tools such as FNA, forceps, and cryoprobe sampling. Experienced-center series using these multimodal workflows report high yields and molecular adequacy, but no randomized trial has established one combination as universally superior. Programs should select a reproducible strategy based on local imaging resources, pathology support, lesion characteristics, operator experience, and safety protocols.

## 11. Advanced and Emerging Applications

### 11.1. Bilateral Same-Session Procedures

RAB enables bilateral same-session lung-nodule sampling, accessing lesions in both lungs during one anesthetic event, a capability generally avoided with TTNB because of bilateral pneumothorax risk [32,33]. Chrissian and colleagues [32] described sampling multiple synchronous nodules during same-session RAB, and Fernandez-Bussy and colleagues [33] evaluated diagnosis of multiple pulmonary nodules, including procedures combined with mediastinal staging. These reports support technical feasibility, but comparative outcome data remain limited.

### 11.2. Aortopulmonary Window and Extended Mediastinal Access

Ion’s fully articulating distal tip can permit navigation into left upper-lobe subsegmental bronchi for CBCT-guided TBNA of the aortopulmonary window (station 5), a region typically inaccessible to standard linear EBUS because of aortic-arch interposition [29]. Published reports also describe robotic access to station 6 and other challenging mediastinal locations [29,30,31]. These reports suggest a potential extension of bronchoscopic nodal staging, although the evidence remains limited to case reports and early technical experience.

### 11.3. Single Anesthetic Diagnosis and Resection

The single-anesthetic bronchoscopy and resection (SABR) paradigm—in which RAB biopsy with intraoperative frozen pathology confirmation is immediately followed by robotic-assisted thoracoscopic resection in the same anesthetic event—has compelling time-to-treatment and cost implications. Weiser and colleagues [51] compared 40 SABR patients to 30 staggered-procedure controls: significant reduction in time from clinic to operating room, lower total costs, and comparable intraoperative and postoperative outcomes. This model eliminates the diagnostic-to-resection delay that adversely affects lung cancer survival and is particularly attractive for patients with small peripheral nodules at high clinical probability for early-stage malignancy.

### 11.4. Fiducial Marker Placement and Preoperative Localization

RAB provides a highly precise platform for intrapulmonary fiducial marker deployment to guide SBRT targeting or surgical localization. Benn and colleagues [71] described indocyanine green-soaked fiducial marker deployment via RAB prior to VATS for nodule localization—demonstrating precise and reliable airway-based marker placement with pneumothorax risk substantially below CT-guided percutaneous fiducial placement.

### 11.5. Transbronchial Ablation: Therapeutic Horizons

The convergence of precise robotic navigation with miniaturized energy delivery enables bronchoscopic therapeutic ablation of early-stage peripheral malignancies. Microwave ablation (MWA) via bronchoscopic delivery is the most advanced under investigation. De Leon and colleagues [72] demonstrated the safety of bronchoscopic MWA in swine peripheral lung using RAB; ongoing clinical trials evaluate this for curative-intent treatment of stage IA lung cancer in patients ineligible for surgery or SBRT, as well as bridging therapy. Diffusing alpha-emitter radiation therapy (DaRT), cryoablation, and vapor thermal ablation are additional modalities in early bronchoscopic therapeutic investigation. If validated, RAB could transform from a diagnostic tool into a comprehensive one-stop interventional hub for early-stage lung cancer.

## 12. Learning Curve, Training, and Competency

Skill acquisition in RAB varies among operators. In a learning-curve analysis from Memorial Sloan Kettering, Bott and colleagues [52] analyzed RAB procedures performed by 9 proceduralists (interventional pulmonologists and thoracic surgeons) over their first 50 cases each. Median procedure time decreased from 62 min during the first 10 cases to 39 min after case 40 (*p* < 0.001). Approximately half of the proceduralists achieved technical proficiency within 25 lesions, and operators increasingly targeted more challenging lesions after the initial phase.

The AABIP-IASLC guideline [64] recommends ongoing program-level quality monitoring, including strict diagnostic yield, molecular adequacy, complications, and time-to-treatment. Intuitive’s FDA clearance for AI-integrated navigation [18] may provide navigational decision support, but its effect on learning-curve variability and clinical outcomes remains unproven.

## 13. Cost-Effectiveness and Healthcare Value

A decision-analytic model [73] quantified the economic value of improved bronchoscopy sensitivity for malignancy, demonstrating that each percentage-point increase in diagnostic sensitivity generates substantial downstream health and cost savings by reducing non-diagnostic procedures, repeat invasive testing, and delayed treatment initiation—supporting the economic case for RAB even at higher per-procedure cost.

The comparative cost-effectiveness of RAB vs. TTNB is particularly favorable when accounting for downstream costs of pneumothorax management (disproportionately high in emphysematous patients), repeat procedures following non-diagnostic TTNB, and the system cost of delayed diagnosis and later-stage treatment. The SABR paradigm generates additional savings by eliminating a separate diagnostic admission and compressing the pre-treatment diagnostic interval [51]. Capital investment is substantial: approximately $500,000–$700,000 for the robotic platform and $250,000–$350,000 for mobile CBCT, although pricing varies by vendor, configuration, and site. Reimbursement under Medicare and most commercial payers is available under navigational bronchoscopy CPT codes when clinical indications are met. The downstream revenue generated by RAB programs—through medical oncology, thoracic surgery, radiation oncology, and molecular diagnostic referrals—substantially exceeds procedural reimbursement alone and should be incorporated into institutional business case analyses.

## 14. Future Directions and Emerging Technologies

### 14.1. Artificial Intelligence Integration

AI is poised to transform multiple RAB workflow facets. In October 2025, Intuitive received FDA clearance for AI integration across Ion’s complete navigational workflow—the first AI-embedded bronchoscopy navigation system [18]. A randomized controlled trial by Agbontaen and colleagues [74] demonstrated AI-guided bronchoscopy instruction superior to expert human instruction for critical care physicians—illustrating AI’s potential to reduce learning curve variability and democratize RAB training broadly. Future applications include automated CT airway segmentation, AI-assisted nodule risk stratification, real-time AI-guided navigation path optimization, intraoperative machine learning-based endobronchial lesion detection, and AI-augmented ROSE cytology interpretation.

### 14.2. Optical Biopsy: Needle-Based Confocal Laser Endomicroscopy

Needle-based confocal laser endomicroscopy (nCLE) represents an emerging optical biopsy technology providing real-time histologic information via fluorescence-based needle probes during RAB. Manley and colleagues [75] demonstrated that nCLE during RAB could identify diagnostic optical patterns correlating with malignancy, potentially enabling rapid on-site optical characterization before formal tissue sampling—a ‘real-time virtual biopsy’ capability that may further reduce non-diagnostic procedure rates.

### 14.3. Next-Generation Platform Development

Ongoing platform development targets: sub-3 mm outer diameter catheters for deeper airway access; integrated real-time CBCT without separate units; closed-loop robotic feedback for automated biopsy tool advancement; single-use disposable designs reducing per-case cost and sterilization logistics; and expanded therapeutic capabilities including miniaturized ablation systems, brachytherapy seed placement, and direct intratumoral immunotherapy delivery. The Galaxy System’s integrated TiLT represents a first step toward self-contained CTBD correction without separate CBCT infrastructure, which may eventually expand access to intraoperative localization beyond major academic centers.

### 14.4. Geographic Access and Equity

Realizing RAB’s population-level impact requires addressing geographic access inequities. RAB is currently concentrated at tertiary academic centers and large community cancer programs in metropolitan areas and remains unavailable to many rural and geographically isolated populations. The Bonn University Group’s first 50-case Ion series [76] and reports from European and Asian centers document broader adoption. Real-time remote expert supervision (tele-proctoring) to support credentialing and quality at lower-volume or rural centers, regional center-of-excellence credentialing, and structured outreach programs are potential strategies to extend RAB access.

## 15. Integration with Lung Cancer Screening Programs

The expansion of LDCT screening is increasing the number of PPLs requiring risk stratification and tissue evaluation. Lung-RADS v2022 provides a standardized framework for managing screen-detected nodules [26]. As screening programs expand, demand for tissue sampling of Lung-RADS 4A and 4B nodules may strain existing diagnostic capacity, including TTNB services, and bronchoscopic approaches such as RAB may help meet this demand [1,26].

RAB may be particularly useful for screening-detected lesions when concurrent mediastinal staging, bronchoscopic access to multiple nodules, or avoidance of transthoracic pneumothorax risk is important. It should not, however, be considered the automatic procedural modality for every screening-detected PPL: TARGET’s strict yield was 61.6%, and modality selection should reflect lesion anatomy, malignancy probability, patient risk, and local expertise [21,46]. Implementation barriers include workforce limitations, capital and infrastructure requirements, geographic inequity, and evolving reimbursement. Programs should monitor strict diagnostic yield, molecular adequacy, complications, and time-to-treatment.

## 16. Limitations, Controversies, and Evidence Gaps

Despite the advances documented throughout this review, several limitations should inform clinical decision-making and future research. Many studies originate from high-volume centers with access to advanced imaging, which may limit generalizability to broader practice settings.

### 16.1. Study Design and Evidence Quality

Most RAB evidence derives from observational studies, single-arm prospective cohorts, and retrospective series. VERITAS [17] established non-inferiority of navigational bronchoscopy to TTNB for 12-month diagnostic accuracy, with fewer pneumothoraces, but evaluated navigational bronchoscopy broadly rather than RAB alone and was conducted at expert centers. RELIANT [49] established non-inferiority, not superiority, of RAB to ENB. The ERS 2025 RAB + CBCT comparison remains a non-peer-reviewed conference abstract [40]. In addition, literature screening for this narrative review was conducted by a single author without duplicate study selection or formal risk-of-bias assessment, creating potential selection and confirmation bias. SANRA improves reporting transparency but does not eliminate these limitations.

### 16.2. Heterogeneity in Diagnostic Yield Reporting

Substantial heterogeneity exists in diagnostic-yield definitions, procedural techniques, patient populations, and adjunctive imaging. The range from TARGET’s 61.6% adjudicated strict yield to much higher intermediate or single-center estimates reflects methodological differences as much as technical performance. The ATS/ACCP statement recommends strict yield and recommends against intermediate or liberal definitions [45]. Many studies predate that consensus, and adherence remains incomplete. The Ali meta-analysis reported substantial heterogeneity (I^2^ = 65.6%) [8]. Contemporary interpretation should therefore prioritize strict estimates, report intermediate values only with explicit labeling, and avoid pooling unlike definitions. CBCT improves tool-in-lesion confirmation, but diagnostic-yield benefit remains mixed and platform-dependent [36,46,50].

### 16.3. Operator Experience, Institutional Volume, and Generalizability

RAB outcomes are influenced by operator experience and institutional procedural volume, raising concerns about generalizability to community and lower-volume settings. In the learning-curve analysis by Bott and colleagues [52], approximately half of proceduralists achieved proficiency within 25 lesions, and diagnostic yield at this high-volume academic center ranged from 58% to 83% across operators; other learning-curve studies have reported similar operator-dependent improvement [77,78,79]. Most published series still come from experienced centers, so the performance of RAB in lower-volume settings remains incompletely defined.

### 16.4. Infrastructure Requirements and Access Inequity

General anesthesia, advanced bronchoscopy infrastructure, and access to intraprocedural imaging create substantial and unevenly distributed barriers. Fixed CBCT requires a purpose-built hybrid suite, while mobile CBCT adds capital cost, workflow demands, and radiation exposure. Rural and geographically isolated systems may have access to a robotic platform without CBCT or specialized pathology support. Because published high-yield multimodal series frequently originate from expert centers, their results may not be reproducible in lower-resource settings, and CBCT should not be assumed to improve diagnostic yield uniformly [46,50].

### 16.5. Emerging Technologies: Limited Evidence Base

Randomized trials now support improved diagnostic yield with the 1.1 mm cryoprobe [60,63], including a dedicated PPL trial [60]. Safety findings are not uniform: FROSTBITE-2 reported no pneumothorax requiring chest-tube placement in the cryoprobe arm and no grade 3–4 bleeding in either arm [63], whereas Cryo-RCT found more moderate bleeding and pneumothorax with cryobiopsy [60]. Neither trial was restricted to robotic bronchoscopy, and neither evaluated a complete RAB + CBCT workflow. Evidence supporting multimodal RAB + CBCT + cryobiopsy therefore remains largely observational. AI-assisted Ion navigation and transbronchial ablation also remain emerging technologies without sufficient comparative outcome evidence to support claims of clinical superiority.

### 16.6. Cost-Effectiveness Data Gaps

Formal cost-effectiveness analyses comparing RAB with competing modalities remain limited and largely institution-specific. The decision-analytic model by Ost and colleagues [73] provides a useful framework but relies on assumptions about improvements in diagnostic sensitivity that may not be universally reproducible. The single-anesthetic biopsy and resection cost data reported by Weiser and colleagues [51] are derived from a small single-center series and require multicenter validation before they inform institutional investment decisions. In addition, current CPT coding does not distinguish RAB performed with versus without CBCT, which may discourage investment in higher-capital imaging approaches.

## 17. Conclusions

RAB is an important advance in the evaluation of PPLs, offering navigational reach, catheter stability, and compatibility with multimodal localization and tissue acquisition. Its diagnostic performance should be described using strict ATS/ACCP definitions rather than headline intermediate estimates. CBCT reliably improves tool-in-lesion confirmation, and cryobiopsy can improve tissue acquisition, but neither adjunct provides a uniform diagnostic-yield benefit across all platforms, centers, or patients [36,45,50,60,63].

Using the recommended strict definition, the most comprehensive meta-analysis reported a pooled RAB yield of 69.6%, and TARGET reported 61.6% [10,21]. Intermediate pooled estimates of approximately 84–87% are substantially higher but are not the preferred benchmark, because the ATS/ACCP statement recommends against intermediate and liberal definitions [8,10,45]. VERITAS showed that navigational bronchoscopy as a category was non-inferior to TTNB for 12-month diagnostic accuracy, with fewer pneumothoraces [17]; it did not establish RAB-specific superiority.

Outcomes remain dependent on lesion characteristics, sampling tools, operator experience, procedural volume, and imaging infrastructure. TARGET and a 2026 clinical review of pulmonary nodules provide an important counterweight to high-yield single-center reports: strict multicenter performance may be similar to other guided bronchoscopic approaches, and localization adjuncts have produced mixed yield results [21,46]. Future studies should use strict yield, distinguish yield from diagnostic accuracy, and prospectively evaluate the incremental value, radiation exposure, and safety of adjunctive imaging and cryobiopsy.

Beyond diagnosis, RAB is expanding the bronchoscopic frontier into same-session bilateral sampling, combined peripheral biopsy and mediastinal staging, fiducial marker placement, single-anesthetic biopsy-and-resection workflows, and investigational transbronchial therapeutic ablation. RAB is likely to play an increasing role within multidisciplinary lung cancer pathways as evidence continues to evolve.

## Figures and Tables

**Figure 1:**
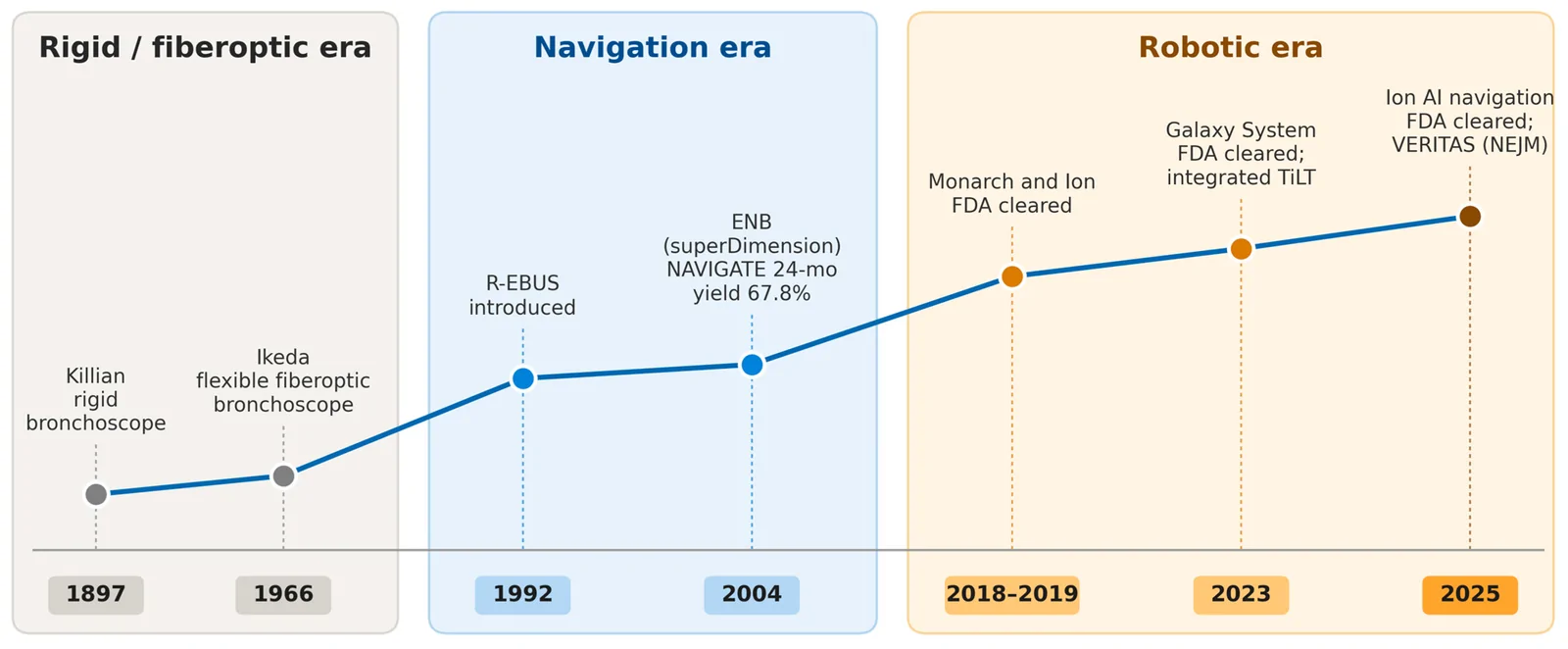
Timeline of bronchoscopy technological evolution: from Killian’s 1897 rigid bronchoscopy through flexible fiberoptic bronchoscopy (Ikeda, 1966), radial endobronchial ultrasound (1992), electromagnetic navigation bronchoscopy (2004) and the NAVIGATE 24-month results [6], FDA clearance of Monarch and Ion (2018–2019) [14,15], the Galaxy System with integrated Tool-in-Lesion Tomosynthesis (TiLT) (2023) [16], and FDA clearance of AI-integrated Ion navigation and publication of the VERITAS trial (2025) [17,18]. The ascending line is a schematic depiction of technological evolution only; vertical position does not represent diagnostic yield, and the figure does not establish monotonic improvement in yield across eras. AI = artificial intelligence; ENB = electromagnetic navigation bronchoscopy; R-EBUS = radial endobronchial ultrasound.

**Figure 2:**
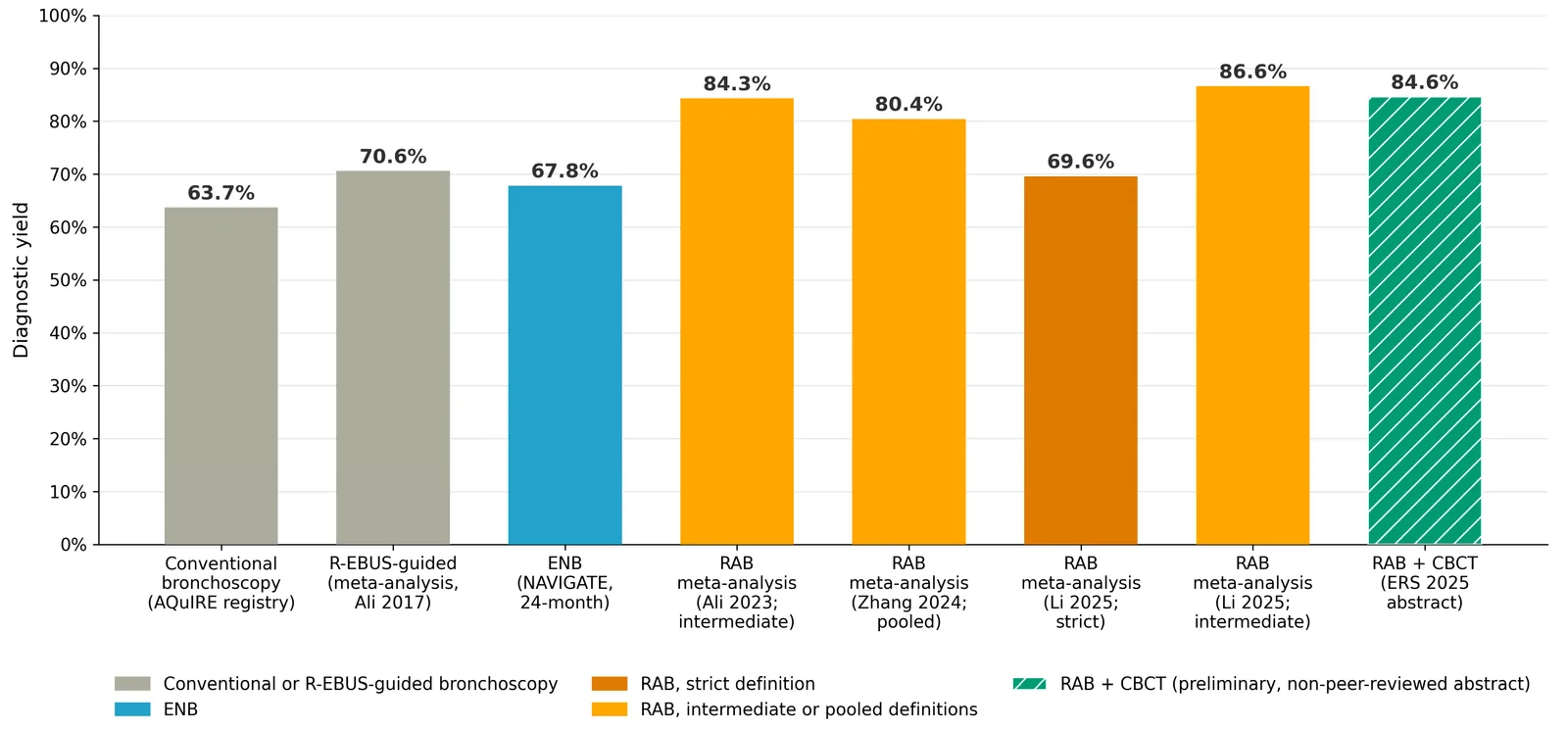
Diagnostic yield by bronchoscopic modality across selected registries, meta-analyses, and trials [4,5,6,8,9,10,40]. Values are not directly comparable because study design, lesion characteristics, and yield definitions differ. The ATS/ACCP statement recommends a strict definition and recommends against intermediate or liberal definitions as primary yield measures [45]. The ERS 2025 RAB + CBCT result is preliminary, non-peer-reviewed conference-abstract evidence and is shown in a separate color. ATS/ACCP = American Thoracic Society/American College of Chest Physicians; CBCT = cone-beam CT; ENB = electromagnetic navigation bronchoscopy; ERS = European Respiratory Society; RAB = robotic-assisted bronchoscopy; R-EBUS = radial endobronchial ultrasound.

**Figure 3:**
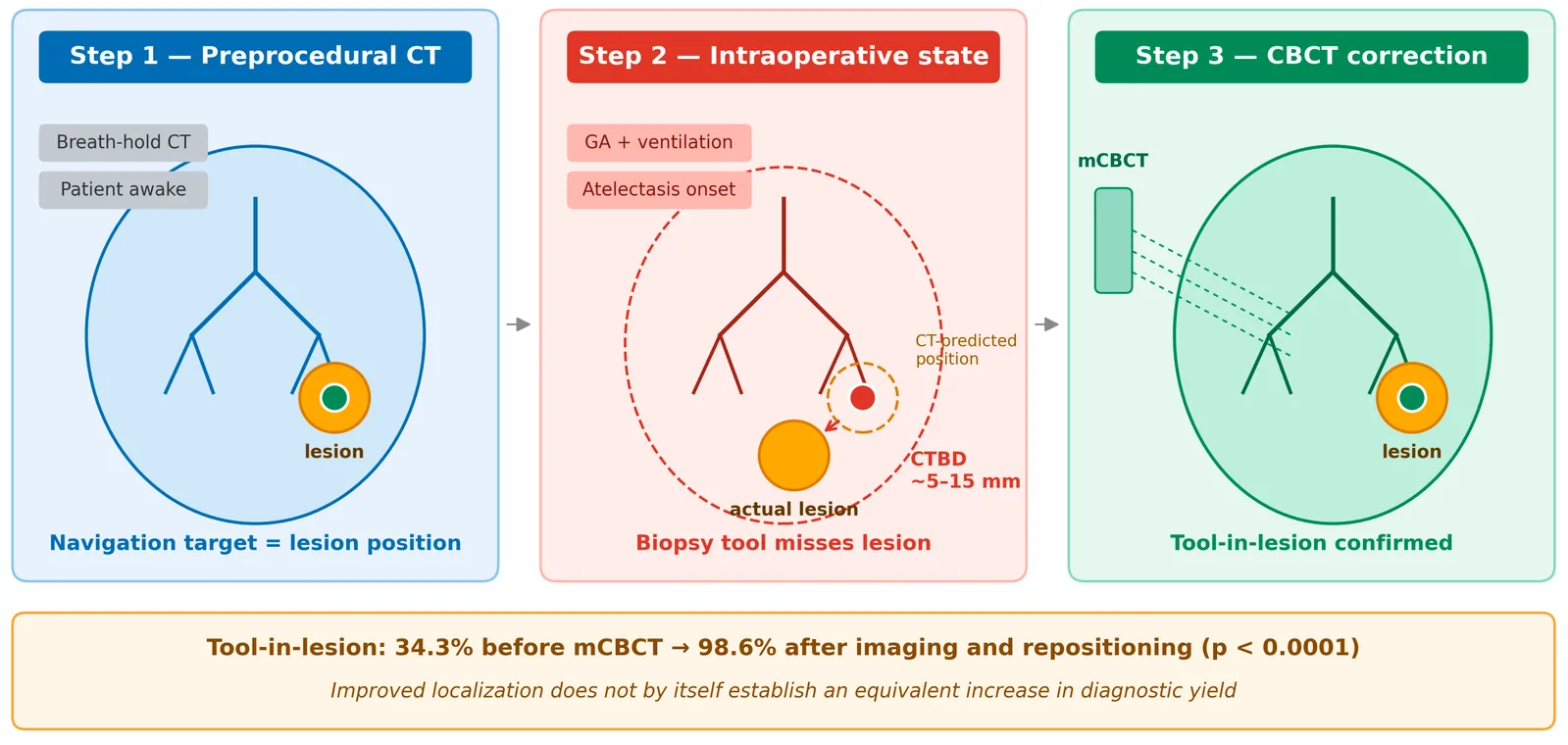
Schematic illustration of CTBD and mobile cone-beam CT correction during robotic-assisted bronchoscopy. In the study by Bashour and colleagues, tool-in-lesion confirmation increased from 34.3% to 98.6% after mobile CBCT imaging and repositioning [36]. CBCT = cone-beam CT; CTBD = CT-to-body divergence; GA = general anesthesia; mCBCT = mobile CBCT.

**Table 1:** Comparative features of FDA-cleared robotic-assisted bronchoscopy platforms.

Feature	Ion™ (Intuitive Surgical)	Monarch™ (J&J/Auris)	Galaxy System™ (Noah Medical)	Clinical Implication
FDA Clearance	February 2019	March 2018	March 2023	Ion/Monarch: 6+ yrs evidence; Galaxy: emerging
Navigation	Shape-sensing (fiber-optic Bragg gratings)	Electromagnetic navigation (CASE system)	Electromagnetic + integrated TiLT tomosynthesis	Shape-sensing: no metal interference; TiLT: self-contained CTBD correction
Scope OD	3.5 mm single catheter	Outer: 6 mm; Inner: 4.2 mm (dual scope)	4.0 mm single scope	Ion: greatest distal reach (6–7th gen airway)
Working Channel	2.0 mm	2.1 mm (inner scope)	2.1 mm	All accept 1.1 mm cryoprobe; Ion accepts PeriView FLEX needle
Vision During Biopsy	No (vision probe removed)	Yes (integrated camera)	Yes (integrated camera)	Monarch/Galaxy allow real-time visual confirmation during sampling
Imaging Integration	Compatible with mobile and fixed CBCT (e.g., Siemens Cios Spin; GE Healthcare)	Compatible with fluoroscopy and CBCT; no integrated system	Integrated TiLT+ digital tomosynthesis + augmented fluoroscopy	Ion CBCT: highest evidence base; Galaxy: self-contained CTBD solution
Scope Reuse	Reusable (manufacturer reprocessed)	Reusable (manufacturer reprocessed)	Single-use disposable	Galaxy eliminates reprocessing requirements; comparative per-case cost data are limited.
AI Integration	FDA-cleared AI navigation (October 2025)	None announced	None announced	Ion: first AI-embedded bronchoscopy navigation system
Key Trial	PRECIsE, RELIANT	BENEFIT, TARGET (*n* = 679)	FRONTIER (*n* = 19 nodules in 18 patients)	TARGET: largest prospective multicenter single-arm RAB study; FRONTIER: early first-in-human feasibility study
Reported Diagnostic Yield (definition as published)	~84–91% with CBCT (strict and intermediate definitions)	61.6% adjudicated strict yield; malignancy sensitivity 78.8% (TARGET)	89.5% strict; 94.7% intermediate (FRONTIER)	TARGET values reflect the final primary publication; definitions differ across studies, and no cross-platform ranking should be inferred.

All three platforms share preprocedural CT-based path planning, robotic actuation, and compatibility with standard biopsy tools. Yield values are reported using the definitions applied in the source studies, which include strict and intermediate estimates; definitions differ across studies, and no cross-platform ranking should be inferred. CBCT = cone-beam CT; CTBD = CT-to-body divergence; OD = outer diameter; TiLT = Tool-in-Lesion Tomosynthesis.

**Table 2:** Clinical factors influencing patient selection for robotic-assisted bronchoscopy.

Category	Clinical Scenario for RAB	Evidence Base/Rationale	Certainty of Evidence and Basis
Primary indication: PPL location and access	Peripheral pulmonary lesion (PPL) in the outer two-thirds of the lung where conventional flexible bronchoscopy has limited diagnostic yield	Conventional bronchoscopy yield: 14% for peripheral <2 cm lesions; 31% for inner two-thirds lesions [22]. RAB accesses 6th–7th-generation airways with navigation success >95% [14,23]. ACCP/NCCN guidelines [24,25]: prefer the least invasive approach enabling simultaneous diagnosis + staging.	Moderate certainty—supported by multiple prospective cohorts and endorsed by ACCP/NCCN for a bronchoscopic-first approach; underlying data largely non-randomized
Primary indication: Nodule malignancy risk	Intermediate- to high-probability pulmonary nodules (Lung-RADS 4A/4B; Mayo model intermediate–high risk; 5–65% probability) requiring tissue diagnosis	ACCP guidelines: intermediate-risk nodules (5–65% malignancy probability per Mayo model) warrant tissue sampling [24]. Lung-RADS category 4A and 4B nodules warrant PET-CT and/or tissue sampling according to size, growth, and features [26].	Moderate certainty—guideline consensus (ACCP/NCCN/ACR Lung-RADS) that intermediate/high-risk nodules warrant tissue; RAB is a reasonable first option when a bronchoscopic approach is appropriate
Primary indication: Molecular profiling requirement	Suspected or known NSCLC requiring tissue for comprehensive molecular profiling: NGS panel, PD-L1 IHC, ALK/ROS1 FISH, TMB—particularly when liquid biopsy is insufficient or tissue confirmation of driver mutation needed	ACCP/NCCN [24,25]: obtain genomic alterations and PD-L1 status at diagnosis and re-biopsy at disease progression. ssRAB cryobiopsy yields 100% molecular adequacy in the Oberg series [27]; NGS success 96% in MSK series [28]. Tissue architecture preserved with cryoprobe—essential for IHC interpretation.	Low certainty—molecular profiling is guideline-required for NSCLC; RAB + cryobiopsy molecular adequacy reported from observational series
Primary indication: Simultaneous mediastinal staging	Peripheral lesion with FDG-avid mediastinal/hilar lymphadenopathy on PET-CT or enlarged nodes on CT where combined peripheral biopsy + mediastinal EBUS-TBNA in a single procedure is clinically appropriate	ACCP/NCCN: diagnose the primary lesion and stage in a single procedure when feasible [24,25]. RAB enables same-session EBUS-TBNA and peripheral biopsy. Published station 5/6 reports describe aortopulmonary window access and other challenging mediastinal locations with robotic bronchoscopy [29,30,31]. Chrissian and Fernandez-Bussy reports support bilateral and multi-site same-session RAB feasibility [32,33].	Low certainty—single-procedure diagnosis and staging is endorsed in principle; the combined RAB workflow is supported by observational experience
Potential advantage over CT-TTNB: Elevated pneumothorax risk	Patients with emphysema, bullous lung disease, or hyperinflated lungs (pneumothorax risk driven by emphysema severity and distribution along the needle path rather than a COPD diagnosis or spirometric threshold per se) where CT-guided TTNB carries prohibitive pneumothorax risk	CT-TTNB pneumothorax rate 20–25%; tube thoracostomy 7–8% overall; risk substantially higher with emphysematous parenchyma overlying biopsy path [2]. RAB: pooled PTX 2.0%; tube 0.5% [10]. RAB transbronchial approach avoids traversal of emphysematous parenchyma and visceral pleura entirely.	Low certainty—a lower pneumothorax risk than CT-TTNB is inferred from pooled observational series; no randomized comparison exists
Potential advantage over CT-TTNB: Contralateral lung compromise	Patients with single functional lung, severe contralateral lung disease, or severely reduced pulmonary reserve where a pneumothorax would be immediately life-threatening	TTNB pneumothorax rate 20–25%; in single-lung patients, any pneumothorax may be clinically consequential. RAB pneumothorax rates are approximately 2.0% and are usually managed conservatively [8,10]. Consensus documents include the Chinese expert consensus [34] and the World Expert Consensus [35]; neither provides comparative outcomes specific to patients with a single functional lung.	Very low certainty—expert-consensus rationale only; no comparative outcome data exist for this specific population
Potential advantage over CT-TTNB: Coagulopathy or anticoagulation	Patients with thrombocytopenia (platelets < 50,000), coagulopathy (INR > 1.5), or therapeutic anticoagulation where TTNB hemorrhage risk is elevated and cannot be safely interrupted	TTNB hemorrhage rates are 2–5% at baseline [2]; substantially higher with uncorrectable coagulopathy. RAB hemorrhage rate <0.5% pooled [10]; transbronchial approach avoids major pleural vessel injury. Note: cryobiopsy may have slightly higher endobronchial bleeding risk; balloon blocker precautions may be considered.	Very low certainty—expert opinion;. (expert opinion; not graded)—a transbronchial route avoids pleural-vessel traversal, but comparative data specifically in coagulopathic patients are lacking; TTNB is contraindicated when coagulopathy cannot be corrected
Potential advantage over CT-TTNB: Lesion characteristics limiting TTNB	Deep-seated lesions requiring long parenchymal traversal (>3 cm depth from pleural surface); lesions adjacent to major vascular structures on CT; prior ipsilateral pleurodesis limiting lung mobility; post-pneumonectomy with contralateral lesion	TTNB complication risk correlates with traversal path length, vascular proximity, and pleural adhesions [2]. RAB not affected by pleural factors; navigates intraluminally regardless of parenchymal depth or pleural status.	Low certainty—anatomic rationale; RAB may be favored where CT-TTNB is technically hazardous or impossible; limited direct comparative data
Specialized indication: Bilateral synchronous nodules	Patients with bilateral pulmonary nodules requiring tissue evaluation from both lungs in a single session (e.g., synchronous primaries, screening-detected multifocal nodules, suspected metastatic evaluation from prior extrathoracic malignancy)	Bilateral same-session TTNB is generally avoided. RAB enables bilateral same-session sampling in single anesthetic event [32,33]. Reduces anesthetic events, time-to-diagnosis, and patient travel burden—particularly relevant in geographically isolated populations.	Low certainty—single-session bilateral sampling is a capability of bronchoscopic approaches, including RAB; supported by observational reports
Specialized indication: Lung cancer screening population	LDCT-detected pulmonary nodules in lung cancer screening program participants with Lung-RADS 4A or 4B classification where tissue sampling is indicated per ACR/ACCP/NCCN guidelines	2021 USPSTF expansion [1]: 50–80 years, ≥20 pack-years. Pooled RAB yield ~78% for lesions <2 cm, the size range of most LDCT-detected lesions, using non-strict definitions [9]. Lung-RADS 4B (≥15 mm or growing): tissue sampling recommended [26].	Low certainty—guideline consensus that Lung-RADS 4 nodules warrant tissue evaluation; RAB use in this population is observational
Relative contraindication: Lesion size <8 mm	Very small lesions (<6–8 mm) where tissue yield may be inadequate regardless of navigation success, and clinical management would not change based on histologic results (i.e., continued surveillance planned per Fleischner/Lung-RADS regardless of biopsy result)	Meta-analyses show that lesion size >20 mm predicts higher yield; lesions ≤10 mm had a reported yield of 66.6% even with CBCT [36,37,38]. The UK ssRAB series included lesions 6–30 mm and reported an overall diagnostic yield of 85% [39]. mCBCT may improve small-lesion localization. If a biopsy result will not change management, the procedure is not indicated regardless of technical feasibility.	Case-by-case—not an absolute contraindication; the decision depends on whether a histologic result will change management versus continued surveillance
Relative contraindication: Pure ground-glass opacity (GGO)	Pure GGOs on CT without a solid component where clinical management is likely surveillance per Fleischner/Lung-RADS guidance, or where tissue yield may be insufficient due to low tumor cell density	Pure GGOs may have lower cellular yield. Preliminary, non-peer-reviewed ERS 2025 conference-abstract data included 27.6% pure GGOs and reported 84.6% overall yield with RAB + CBCT [40]; this estimate should not be treated as definitive. Fleischner and Lung-RADS surveillance recommendations remain primary when biopsy would not change management.	Case-by-case. Pure GGO is a lower-yield scenario, not an absolute contraindication. The ERS estimate is preliminary conference evidence; where biopsy is indicated, cryobiopsy may improve circumferential sampling and tissue architecture.
Relative contraindication: Inability to tolerate general anesthesia	Patients with severe cardiovascular or pulmonary comorbidities where general anesthesia and neuromuscular blockade are deemed prohibitively high risk by anesthesia evaluation	RAB is generally performed under general anesthesia with endotracheal intubation and NMB at high-volume centers for optimal CTBD minimization and CBCT integration [41,42]. Moderate sedation is practiced at select centers for straightforward larger lesions (>2 cm, bronchus sign present) but substantially increases CTBD. If GA is not safely feasible: CT-guided TTNB under monitored sedation, or multidisciplinary discussion regarding risk-benefit.	Case-by-case—not an absolute contraindication; moderate sedation may suit select favorable-anatomy cases; multidisciplinary risk–benefit assessment advised [41,42]
Absolute contraindication: Active bronchospasm or severe uncontrolled asthma	Active bronchospasm, status asthmaticus, or severe uncontrolled reactive airways disease precluding safe bronchoscopy with general anesthesia and positive pressure ventilation	Standard bronchoscopy contraindication applies regardless of platform; active bronchospasm significantly increases complication risk under GA with positive pressure ventilation and PEEP. Procedure should be deferred until airway disease is optimally managed.	Contraindication—a general bronchoscopy/anesthesia contraindication (not RAB-specific); defer until bronchospasm is resolved
Absolute contraindication: Hemodynamic instability or acute respiratory failure	Hemodynamically unstable patients, those requiring escalating vasopressor support, or patients in acute respiratory failure dependent on mechanical ventilation or high-flow oxygen support	Standard bronchoscopy/anesthesia absolute contraindication. RAB is an elective diagnostic procedure; should not be performed in acutely ill, unstable patients regardless of clinical urgency of tissue diagnosis.	Contraindication—a standard contraindication to elective diagnostic bronchoscopy; stabilization is required first
Contraindication to cryobiopsy: Uncorrectable severe coagulopathy	Uncorrectable severe coagulopathy (platelets <20,000 or INR > 3.0) if cryobiopsy is planned; standard forceps/FNA biopsy may still be feasible with moderate coagulopathy	Cryobiopsy: larger-core specimen with theoretically higher endobronchial hemorrhage risk. Balloon blocker recommended by some operators for peripheral cryobiopsy. Severe uncorrectable coagulopathy: avoid cryobiopsy; FNA/forceps biopsy with coagulopathy correction is acceptable for standard RAB biopsy.	Case-by-case for cryobiopsy; contraindicated with severe uncorrectable coagulopathy—FNA/forceps acceptable after moderate correction

This table uses descriptive categories that reflect the clinical scenario (Category) and the certainty of the supporting evidence (Certainty of Evidence and Basis); it does not assign formal recommendation strength, and no formal grading system (e.g., GRADE) was applied. Certainty descriptors (moderate, low, very low) reflect study design and directness and are distinct from any recommendation; most supporting data are observational, indirect, or consensus-based. Statements resting on mechanism or clinical reasoning are identified as expert opinion. ACCP = American College of Chest Physicians; ACR = American College of Radiology; CBCT = cone-beam CT; CTBD = CT-to-body divergence; EBUS-TBNA = endobronchial ultrasound-guided transbronchial needle aspiration; FDG = fluorodeoxyglucose; FISH = fluorescence in situ hybridization; FNA = fine-needle aspiration; GA = general anesthesia; GGO = ground-glass opacity; IHC = immunohistochemistry; INR = international normalized ratio; LDCT = low-dose CT; mCBCT = mobile CBCT; MSK = Memorial Sloan Kettering; NCCN = National Comprehensive Cancer Network; NGS = next-generation sequencing; NMB = neuromuscular blockade; NSCLC = non-small cell lung cancer; PD-L1 = programmed death-ligand 1; PEEP = positive end-expiratory pressure; PET-CT = positron emission tomography-CT; PPL = peripheral pulmonary lesion; PTX = pneumothorax; RAB = robotic-assisted bronchoscopy; ssRAB = shape-sensing RAB; TMB = tumor mutational burden; TTNB = transthoracic needle biopsy; USPSTF = US Preventive Services Task Force.

**Table 3:** Anesthetic and ventilatory strategies to optimize procedural success in robotic-assisted bronchoscopy.

Strategy	Mechanism/Protocol	Evidence/Trial	Certainty of Evidence and Basis
General anesthesia with ETT + NMB	Full neuromuscular blockade eliminates respiratory motion artifact during navigation and CBCT; enables controlled PEEP application	Universally adopted at high-volume centers; supported by TARGET and PRECIsE protocols	Low certainty—prospective RAB protocols; no randomized anesthetic comparison.
Individualized PEEP (commonly 8–12 cmH_2_O)	Selected according to target location, body habitus, respiratory mechanics, oxygenation, and imaging response; aims to prevent absorption atelectasis, maintain lung volume, and reduce CT-to-body divergence	Pritchett et al. and related lung-navigation protocols [7]; target-location-dependent expert practice	Low certainty—procedural series and expert protocols.
VESPA protocol (ventilatory strategy to prevent atelectasis)	Standardized lung-protective ventilation: PEEP 8–10 cmH_2_O, TV 6–8 mL/kg IBW, FiO_2_ ≤ 50%, breath-hold during CBCT spin	VESPA multicenter RCT [41]: reduced intraoperative atelectasis vs. standard ventilation	Moderate certainty—RCT evidence for reduced atelectasis; indirect for diagnostic yield.
Low FiO_2_ (≤50%)	Reduces absorptive atelectasis by preventing nitrogen washout in dependent lung zones	I-LOCATE trial [42]: atelectasis onset at ~30 min; FiO_2_ reduction attenuates this	Very low certainty—physiologic rationale.
I-LOCATE protocol (time to atelectasis)	Intubation-to-biopsy completion target <30 min; expeditious scope introduction and navigation minimizes atelectasis accumulation	I-LOCATE trial [42]: significant atelectasis develops by 30 min post-intubation under GA	Low certainty—prospective timing data; the <30 min target is an operational inference.
Recruitment maneuvers before CBCT	Sustained inflation (30 cmH_2_O for 30 s) prior to CBCT spin redistributes atelectatic lung; improves lesion visualization and TIL	Described in expert protocols [7]; not yet RCT-tested in isolation	Very low certainty—expert protocols and mechanistic rationale.
Breath-hold during CBCT spin	Paralysis + brief apnea (5–10 s) during CBCT acquisition reduces motion artifact; improves 3D reconstruction quality	Standard technique in hybrid suite CBCT bronchoscopy protocols [43]; the sustained/prolonged breath-hold technique is further discussed by Ratwani et al. [44]	Low certainty—procedural series and widespread protocol use.
Patient positioning (supine, lateral decubitus, or prone; selected cases)	Supine is standard; lateral decubitus or prone positioning may be selected according to target location to redistribute dependent atelectasis and improve lesion visualization or access	Emerging practice; physiologic rationale; limited prospective comparative data	Very low certainty—theoretical basis with limited prospective data.
Avoid excess suction	Prevents mucus plug redistribution and small airway collapse that exacerbates CTBD post-navigation	Expert recommendation [7]	Very low certainty—expert opinion and mechanistic rationale.
Moderate sedation (selected cases)	For straightforward, larger lesions with favorable anatomy; reduces resource utilization; increases CTBD risk	Some high-volume centers; not recommended for CBCT-dependent cases	Very low certainty—selected-center practice with limited comparative evidence.

3D = three-dimensional; CBCT = cone-beam CT; CT = computed tomography; CTBD = CT-to-body divergence; ETT = endotracheal tube; FiO_2_ = fraction of inspired oxygen; GA = general anesthesia; IBW = ideal body weight; I-LOCATE = Incidence and Location of Atelectasis Developed During Bronchoscopy Under General Anesthesia (trial); NMB = neuromuscular blockade; PEEP = positive end-expiratory pressure; RAB = robotic-assisted bronchoscopy; RCT = randomized controlled trial; TIL = tool-in-lesion; TV = tidal volume; VESPA = Ventilatory Strategy to Prevent Atelectasis.

**Table 4:** Selected studies relevant to robotic-assisted bronchoscopy, including historical comparators, prospective studies, randomized controlled trials, and meta-analyses cited in this review.

Trial/Study [Ref.]	Platform	Study Population (Unit as Reported)	Median Lesion Size	Navigation Success	Diagnostic Yield	Key Findings and Clinical Significance
NAVIGATE 24-month [6]	ENB (superDimension)	1215 patients; 37 centers	2.0 cm	~80%	67.8% (24-month)	Largest prospective ENB cohort; established the 70% diagnostic yield ceiling; CTBD identified as primary mechanism of navigational failure; all RAB studies use this as the key historical comparator
AQuIRE Registry [5]	Conventional nonguided bronchoscopy	581 patients (591 bronchoscopies)	Variable	N/A	63.7%	Multicenter real-world nonguided bronchoscopy registry; establishes fundamental baseline diagnostic yield prior to navigational era; direct evidence for need for navigation-assisted approaches
PRECISION-1 (cadaveric proof of concept) [47]	Ion vs. ENB vs. ultrathin bronchoscopy with R-EBUS	Cadaveric model	—	100% vs. 85% vs. 65%	Localization and puncture: 90% vs. 65% vs. 35% (cadaveric)	First prospective head-to-head: RAB vs. ENB vs. conventional bronchoscopy; Ion +35 pp over conventional; established cadaveric proof-of-concept for shape-sensing navigation superiority
REACH Assessment (cadaveric proof of concept) [48]	Monarch	Cadaveric model	—	—	N/A (cadaveric)	Cadaveric feasibility study of the Monarch platform.
BENEFIT multicenter pilot/feasibility study [15]	Monarch	54 patients	2.0 cm	96.2%	74% intermediate	Prospective, multicenter, single-arm pilot/feasibility study; not randomized. Pneumothorax 3.7%; established early human feasibility of electromagnetic-navigation RAB.
PRECIsE lead-in multicenter feasibility study [14]	Ion (ssRAB)	60 subjects; 67 nodules	2.0 cm median largest cardinal diameter	Biopsy completion: 97.0% of nodules	Not assessed	Prospective multicenter, single-arm lead-in study. Diagnostic yield and sensitivity were not assessed; no pneumothorax or airway bleeding was reported.
TARGET Trial [21]	Monarch	679 patients	1.85 cm (IQR 1.35–2.65 cm).	97.5%	61.6% adjudicated strict yield; malignancy sensitivity 78.8%	Largest prospective multicenter single-arm RAB study; not randomized; adverse-event rate 3.8%.
FRONTIER First-in-Human [16]	Galaxy System (TiLT integrated)	19 nodules in 18 patients	2.0 cm (mean)	100% TiLT-confirmed TIL	89.5% strict/94.7% intermediate	First-in-human Galaxy trial; 100% tool-in-lesion localization via integrated TiLT technology; small sample—multicenter trial NCT06056128 ongoing
RELIANT RCT [49]	Ion (ssRAB) vs. ENB	411 patients	~2 cm	Not reported	77.8% vs. 75.5% (RAB vs. ENB)	Single-center pragmatic cluster-randomized trial; ENB incorporated digital tomosynthesis correction; subgroup findings are exploratory.
VERITAS RCT [17]	Navigational bronchoscopy (ENB-based)	234 patients	10–30 mm	Not reported	12-month diagnostic accuracy 79.0% vs. 73.6%	Navigational bronchoscopy was non-inferior to CT-guided TTNB (*p* = 0.003) but not superior (*p* = 0.17). Pneumothorax 3.3% vs. 28.3%; chest tube and/or admission 0.8% vs. 11.5%. The trial evaluated navigational bronchoscopy broadly, not RAB specifically.
mCBCT + Ion (MD Anderson) [36]	Ion + Cios Spin mCBCT	67 patients	1.7 cm (range 0.9–3.0)	TIL: 34.3% RAB alone → 98.6% with mCBCT (*p* < 0.0001)	TIL improved; diagnostic-yield increment was not established by the TIL change alone	Strong localization evidence for Ion + mCBCT, but CBCT yield benefit is not uniform across platforms; a Monarch study found 70.9% vs. 71.7% strict yield [50].
RAB + CBCT vs. conventional bronchoscopy RCT (ERS 2025 conference abstract; non-peer-reviewed) [40]	Ion + Cios Spin vs. ultrathin CB + 2D fluoroscopy	78 patients; 127 PPLs	11 mm [IQR 9–16]	—	84.6% (strict) RAB + CBCT vs. 23.1% ultrathin bronchoscopy (*p* < 0.001; absolute difference 61.5 percentage points)	Preliminary conference-abstract data: the randomized comparison reported higher yield with RAB + CBCT, but the findings remain non-peer-reviewed and should be interpreted cautiously pending full publication.
Meta-Analysis [8]	Ion + Monarch	1779 lesions; 20 studies	—	—	84.3% intermediate yield (95% CI 81.1–87.2%); I^2^ = 65.6%	Intermediate-definition estimate, not the ATS/ACCP-preferred strict benchmark; PTX 2.3%, chest tube 1.2%, hemorrhage 0.5%; substantial heterogeneity.
Meta-Analysis [9]	Ion + Monarch	725 lesions; 10 studies	—	—	80.4% pooled; cryo studies 90.0% vs. non-cryo 79.0% (*p* < 0.01)	Observational-study meta-analysis associated cryobiopsy use with higher yield; confounding and heterogeneous definitions prevent treating this as proof of a standard-of-care strategy.
Meta-Analysis [10]	Ion + Monarch	27 cohort studies; >2000 lesions	—	—	69.6% strict (95% CI 61.8–76.8%); 86.6% intermediate (95% CI 83.7–89.2%)	Most comprehensive meta-analysis; the strict estimate is the preferred headline benchmark. The wide strict/intermediate gap illustrates why unlike definitions should not be pooled.
Single-Anesthetic Biopsy + Resection [51]	Ion (ssRAB)	40 SABR vs. 30 staged controls	—	—	Surgical outcomes (not diagnostic yield)	Reduced time from clinic → OR; significantly lower total cost; comparable intraoperative and postoperative outcomes; establishes SABR paradigm as safe and cost-effective for high-probability malignancy cases
Learning Curve Analysis [52]	Ion (ssRAB)	9 operators; 442 patients; 551 lesions	1.9 cm (IQR 1.33–2.80)	N/A	Overall 72% (range 58–83% by operator)	Procedure time: 62 min (first 10 cases) → 39 min (after case 40); ~50% of proceduralists proficient within 25 lesions; important for credentialing program design
Molecular Adequacy [28]	Ion (ssRAB)	128 samples (104 primary lung cancer + 24 metastases)	—	N/A	84% adequate for molecular testing; NGS success 96% (25/26); PCR 94% (49/52); PD-L1 IHC 91%	Largest ssRAB molecular adequacy study; demonstrates bronchoscopic samples are suitable for comprehensive precision oncology workup

2D = two-dimensional; ATS/ACCP = American Thoracic Society/American College of Chest Physicians; CB = conventional bronchoscopy; CBCT = cone-beam CT; CI = confidence interval; CT = computed tomography; ENB = electromagnetic navigation bronchoscopy; ERS = European Respiratory Society; IHC = immunohistochemistry; IQR = interquartile range; mCBCT = mobile CBCT; N/A = not applicable; NB = navigational bronchoscopy; NGS = next-generation sequencing; PCR = polymerase chain reaction; PD-L1 = programmed death-ligand 1; PPL = peripheral pulmonary lesion; PTX = pneumothorax; RAB = robotic-assisted bronchoscopy; RCT = randomized controlled trial; R-EBUS = radial endobronchial ultrasound; SABR = single-anesthetic biopsy and resection; ssRAB = shape-sensing RAB; TIL = tool-in-lesion; TiLT = Tool-in-Lesion Tomosynthesis; TTNB = transthoracic needle biopsy. Cadaveric proof-of-concept rows are labeled as such, and their results should not be compared directly with human clinical yields.

**Table 5:** Additional shape-sensing robotic-assisted bronchoscopy (ssRAB) series for peripheral pulmonary lesions, identified from expert consensus documents [34,35] and subsequent publications.

Study (Year)	Inclusion (Size)	N (Unit)	Diagnostic Yield	Complication Rate	Pneumothorax	Bleeding
Fielding et al. 2019 [53]	10–30 mm	29	79.3% strict/96.6% intermediate	6.9%	0	0
Benn et al. 2021 [54]	n.a.	59	86% intermediate	3.8%	3.8%	0
Simoff et al. 2021 [14]	10–35 mm	60 subjects/67 nodules	Not assessed	3.3% (2/60; neither attributed to ssRAB)	0	0
Reisenauer et al. 2022 [43,55]	10–30 mm	30	93.3% strict	6.25%	0	0
Reisenauer et al. 2022 [43,55]	10–30 mm	270	n.a.	4.5%	3.3%	0.8%
Kalchiem-Dekel et al. 2022 [23]	Not restricted	159	81.7%	3%	1.5%	0
Bashour et al. 2024 [36]	10–30 mm	67	86.6% strict	3.0%	1.5%	0% clinically significant
Xie et al. 2024 [56]	8–30 mm	90	87.8% intermediate	3.3%	1.1%	0
Brownlee et al. 2025 [57]	13–33 mm	1121 biopsies	86.1%	4.8%	3.3%	1%
Chan et al. 2026 [39]	6–30 mm	200	85%	3%	0.5%	1%
Brock et al. 2026 [58]	10–30 mm	43	67.4%	7.0%	0	7.0%

Studies are listed chronologically. Strict and intermediate refer to ATS/ACCP 2024 yield definitions where reported; yields without a label were reported without a specified definition. N uses the sample unit reported by each source (patients, lesions, nodules, or biopsies). ATS/ACCP = American Thoracic Society/American College of Chest Physicians; n.a. = not available.

**Table 6:** Biopsy modalities and sampling techniques in robotic-assisted bronchoscopy.

Tool	Diagnostic Yield	NGS Adequacy	Architecture Preserved	360° Acquisition	Best Use Case
FNA needle (21–22G)	31.5–86.6% (variable)	~49% for NGS (smear); 14% for cell block [61]	No (cytology specimen)	No (single-plane aspiration)	ROSE-compatible; rapid cytology; first-pass tool
Forceps biopsy (standard)	54–86.9%	~29% for NGS; 63% for PD-L1 [61]	Partial (some crush)	No (unidirectional bite)	Endobronchial lesions; concentric R-EBUS lesions
1.1 mm cryoprobe (TBCB)	75–97.2% (RAB series)	100% (all cryo specimens in Oberg series)	Yes (preserved architecture)	Yes—freezes circumferentially	Eccentric/adjacent lesions; subcentimeter nodules; GGO; molecular profiling priority
Bronchial brushing	47–54%	Variable; lower than biopsy	No	No	Supplemental; combination with FNA
Multimodal (FNA + forceps + cryobiopsy)	~90% (Oberg et al. [27], *n* = 120)	~100% for cryo component	Yes (cryo component)	Yes (cryo component)	Used at some high-volume centers; highest reported yields in observational series

Values are approximate ranges from individual RAB series cited in the text [27,28,61,62] and are not pooled estimates; yields vary with lesion characteristics and yield definitions. FNA = fine-needle aspiration; G = gauge; GGO = ground-glass opacity; NGS = next-generation sequencing; PD-L1 = programmed death-ligand 1; RAB = robotic-assisted bronchoscopy; R-EBUS = radial endobronchial ultrasound; ROSE = rapid on-site cytologic evaluation; TBCB = transbronchial cryobiopsy.

**Table 7:** Complications and safety profile of robotic-assisted bronchoscopy.

Modality	Overall Pneumothorax	Chest Tube Required	Significant Hemorrhage	Overall Complication Rate
CT-guided TTNB [2]	20–25%	7–8%	2–5%	~25–30%
Conventional/ENB [5,6]	1–2%	<1%	<1%	1–3%
RAB (pooled meta-analytic data, 2025) [10]	2.0%	0.5%	<0.5%	3.0%
RAB + mCBCT (MD Anderson, *n* = 67) [36]	3%	0%	0%	1.5%
RAB + CBCT RCT * (ERS 2025 conference abstract; non-peer-reviewed) [40]	n.r.	n.r.	n.r.	Similar between arms (rates n.r.)
TARGET Trial—Monarch (*n* = 679) [21]	n.r.	n.r.	n.r.	3.8%
Robotic Cryobiopsy (Oberg et al., *n* = 120) [27]	5.4%	Not reported	0%	~5%
AABIP-IASLC Guideline 2025 [64]—Bronchoscopy vs. Percutaneous	2.0% vs. 20–25%	<1% vs. 7–8%	Comparable	~3% vs. ~27%

Safety findings differ between cryobiopsy trials: Cryo-RCT reported more moderate bleeding and pneumothorax with cryobiopsy [60], whereas FROSTBITE-2 observed no pneumothorax requiring chest-tube placement in the cryoprobe arm and no grade 3–4 bleeding in either arm [63]. * The ERS 2025 row is preliminary, non-peer-reviewed conference-abstract evidence; specific complication rates were not reported, and the row is not directly comparable with peer-reviewed trials. AABIP-IASLC = American Association for Bronchology and Interventional Pulmonology–International Association for the Study of Lung Cancer; CBCT = cone-beam CT; CT = computed tomography; ENB = electromagnetic navigation bronchoscopy; ERS = European Respiratory Society; mCBCT = mobile CBCT; n.r. = not reported; RAB = robotic-assisted bronchoscopy; RCT = randomized controlled trial; TTNB = transthoracic needle biopsy.

## Data Availability

No new data were created or analyzed in this study. Data sharing is not applicable to this article.

## Supplementary Materials

The following supporting information can be downloaded at: https://www.mdpi.com/article/10.3390/diagnostics16193151/s1, Table S1: SANRA checklist.
