# Supplementary material for: Robotic-Assisted Bronchoscopy for Peripheral Pulmonary Lesions: Current Evidence, Clinical Applications, and Future Directions
Source: Diagnostics (Basel). 2026 Sep 28;16(19):3151. doi: 10.3390/diagnostics16193151 (PMC13664037; doi:10.3390/diagnostics16193151)
Supplement: Supplementary file 1 [file diagnostics-16-03151-s001.zip › diagnostics-4271956-supplementary.pdf]

SANRA — Completed Self-Assessment

Scale for the Assessment of Narrative Review Articles (Baethge, Goldbeck-Wood & Mertens, Res Integr Peer Rev 2019;4:5)

Manuscript: Robotic-Assisted Bronchoscopy for Peripheral Pulmonary Lesions — Diagnostics (MDPI)

Each of the six SANRA items is rated 0, 1, or 2 (maximum total 12). This completed self-assessment accompanies the revised manuscript, as requested by the reviewers, and indicates where each criterion is met in the revised text.

#	Item	Score	How the manuscript meets the criterion (with revision notes)
1	Justification of the article's importance for the readership	**2**	The Introduction and Section 15 establish clinical importance — the expansion of low-dose CT screening and the resulting diagnostic bottleneck for peripheral pulmonary lesions — and frame the review explicitly for interventional pulmonology and thoracic oncology readers.
2	Statement of concrete aims or formulation of questions	**2**	The revised (structured) Abstract and Section 1 state the review's scope and objectives; Section 2 pre-specifies the aims, inclusion/exclusion criteria, and narrative-synthesis approach.
3	Description of the literature search	**2**	Section 2.1 reports the databases searched (PubMed/MEDLINE, Embase, Web of Science, Cochrane), the date window (January 2018–March 2026), MeSH and free-text terms, the Boolean strategy, and supplementary trial-registry and conference searches. The revision clarified that SANRA governs the review and PRISMA 2020 was used only for search/screening-flow reporting, and explicitly acknowledged single-author screening as a potential source of selection/interpretation bias (Sections 2.3 and 16.1).
4	Referencing	**2**	Claims are supported by current, relevant primary sources. The revision added the fourth major meta-analysis (Pyarali 2024), both ssRAB consensus statements, additional prospective ssRAB series (new Table 2b), the FROSTBITE-2 randomized cryobiopsy trial, and complication case reports; conference-abstract sources are now flagged as non-peer-reviewed.
5	Scientific reasoning (including balance)	**2**	The revision tempered definitive/promotional language, consistently separated technical feasibility from diagnostic yield from proven comparative benefit, added a neutral modality-selection framework (Section 10.3), expanded the limitations (Section 16), and replaced graded table labels with neutral wording plus a footnote stating that no formal (e.g., GRADE) evidence grading was applied.
6	Appropriate presentation of data	**2**	Diagnostic-yield data are presented against the ATS/ACCP 2024 consensus definitions with confidence intervals where available; results are organized in structured tables and figures, and heterogeneity and non-comparability across studies are explicitly noted.

Total SANRA score: 12 / 12

Scoring anchors (per Baethge et al. 2019): 0 = criterion not met; 1 = partially met; 2 = fully met. This self-assessment is provided for transparency and does not replace editorial or peer judgement.
